# Transcriptome, Plant Hormone, and Metabolome Analysis Reveals the Mechanism of Purple Pericarp Formation in ‘Zihui’ Papaya (*Carica papaya* L.)

**DOI:** 10.3390/molecules29071485

**Published:** 2024-03-27

**Authors:** Xiaming Wu, Min Yang, Chuanhe Liu, Ruibing Kuang, Han He, Chenping Zhou, Yuerong Wei

**Affiliations:** Institute of Fruit Tree Research, Guangdong Academy of Agricultural Sciences, Key Laboratory of South Subtropical Fruit Biology and Genetic Resource Utilization, Ministry of Agriculture and Rural Affairs, Guangdong Provincial Key Laboratory of Tropical and Subtropical Fruit Tree Research, Guangzhou 510640, China; wuxiaming625@126.com (X.W.);

**Keywords:** *Carica papaya* L., transcriptome, metabolome, plant hormone, anthocyanin, purple pericarp

## Abstract

The color of the pericarp is a crucial characteristic that influences the marketability of papaya fruit. Prior to ripening, normal papaya exhibits a green pericarp, whereas the cultivar ‘Zihui’ displays purple ring spots on the fruit tip, which significantly affects the fruit’s visual appeal. To understand the mechanism behind the formation of purple pericarp, this study performed a thorough examination of the transcriptome, plant hormone, and metabolome. Based on the UPLC-ESI-MS/MS system, a total of 35 anthocyanins and 11 plant hormones were identified, with 27 anthocyanins and two plant hormones exhibiting higher levels of abundance in the purple pericarp. In the purple pericarp, 14 anthocyanin synthesis genes were up-regulated, including CHS, CHI, F3H, F3′5′H, F3′H, ANS, OMT, and CYP73A. Additionally, through co-expression network analysis, three MYBs were identified as potential key regulators of anthocyanin synthesis by controlling genes encoding anthocyanin biosynthesis. As a result, we have identified numerous key genes involved in anthocyanin synthesis and developed new insights into how the purple pericarp of papaya is formed.

## 1. Introduction

Papaya (*Carica papaya* L.) is a perennial fleshy herb and a significant fruit-bearing tree in tropical and subtropical regions. It is popular worldwide, being rich in vitamins, protein, catabolic enzymes, and minerals [1]. An important breeding trait is the appearance of papaya pericarp, which is crucial to consumer evaluation. The primary objective of breeding is to select a papaya pericarp’s external traits to meet public expectations [2].

The vibrant colors seen in plant flowers, fruits, and leaves are often a result of the buildup of pigment substances [3]. Natural plant pigments can generally be categorized into four groups: chlorophyll, carotenoids, anthocyanins, and betalains. Chlorophyll is a critical pigment for photosynthesis and is widely distributed in plants, giving plant organs a dark or light green appearance based on chlorophyll content differences [4]. Natural terpenoid lipid-soluble pigments called carotenoids show mainly as yellow, orange, and red colors [5,6]. Plants are widely distributed with anthocyanins, water-soluble pigments that are known for their antioxidant properties and present in blue, purple, and red colors [7,8]. Betalains are primarily found in sugar beets, distinguishing them from the aforementioned three pigments [9]. The presence of these plant pigments contributes to the vibrant and colorful appearance of plants [10].

Anthocyanins are the most significant coloring compounds in flavonoids. Up to now, anthocyanins have been successfully isolated and identified in more than 600 plant types [11]. The mechanism of anthocyanins that allows multiple colors is complicated, mainly involving three aspects: First, the type and concentration of pigments are the primary factors influencing color variation, anthocyanin pigments are found in a wide variety of species and present a variety of colors. The most basic types are pelargonidin (orange), cyanidin (red), and delphinidin (purple). These are further modified through methylation, glycosylation, and acylation to produce different types of anthocyanin glycosides [12]. Pigments of different colors interact to produce a spectrum of colors, and the concentration of pigment directly affects the final color display. For example, pelargonidin typically appears orange, but when accumulated in large amounts, it tends to appear purple-red or black-red, while low concentrations result in a pink hue [3]. Second, altering anthocyanins in different ways will dramatically alter how they render color. When anthocyanins are methylated, the color will clearly change to red [13]. An important stage in the molecular alteration of anthocyanins is glycosylation; the quantity and location of this modification greatly affect how the color appears. They will have a tendency to turn bluish violet due to glycosylated anthocyanins [14]. In addition to glycosylation location, acylation modification is also influenced by the number and position of acyl groups. For instance, polyaromatic acyl modification at positions 3′ and 7′ can cause the plant to appear blue, while acyl modification at positions 3 and 5 results in a purplish-red color [15]. Furthermore, anthocyanin’s color and physicochemical characteristics can be altered by processes such as fatty acid acylation and aromatic acid acylation [14]. Third, the hue of anthocyanins has also been shown to be significantly affected by environmental factors including illumination, temperature, humidity, nutrition, pH, and hormonal stimulation [16,17].

In plants, anthocyanin biosynthesis occurs on the surface of the endoplasmic reticulum as part of the flavonoid pathway. Numerous plants have a highly conserved anthocyanin biosynthesis pathway that has been thoroughly explored in other fruit trees [18]. Chalcone synthase (CHS) is responsible for producing chalcone from anthocyanins, which are typically composed of one 4-coumarin CoA and three malonyl CoAs binding in the cytoplasm [19]. The enzyme chalcone isomerase (CHI) converts chalcone to flavanone naringenin, while flavanone 3-hydroxylase (F3H) catalyzes the formation of dihydrokaempferol (DHK) from flavanone naringenin [20,21]. Conversely, dihydroflavonol reductase (DFR) directly converts DHK into colorless pelargonidin. Further, DHK forms dihydroquercetin (DHQ) by activating dihydroflavonol-3′-hydroxylase (F3′H), while DHQ and DHK form dihydromyricetin (DHM) under the action of dihydroflavonol-3′, 5′-hydroxylase (F3′5′H) [22,23,24]. The four-position carbonyl group is reduced to the hydroxyl group under the action of DFR in DHK, DHQ, and DHM, resulting in colorless anthocyanins [25]. Then, colorless proanthocyanidins are synthesized into three basic anthocyanin types: pelargonidin, cyanidin, and delphinidin by anthocyanidin synthase (ANS) [26]. Anthocyanins that are destabilized are converted to stable anthocyanins by UDP-glucose flavonoid 3-glucosyltransferase (UFGT) [27,28].

‘Zihui’ is a newly developed papaya variety by the Fruit Tree Research Institute of the Guangdong Academy of Agricultural Sciences. It develops a well-established root system, produces high yields, and has good stress resistance, especially in high-temperature environments, where its continuous bearing ability is strong. In comparison to conventional varieties, ‘Zihui’ shows great potential for widespread adoption and use, but the presence of purple ring spots on the fruit tip can negatively impact consumer preference. Therefore, in our study, transcriptome, plant hormones, and metabolome analysis were used to identify key genes involved in anthocyanin synthesis and to develop new insights into how the purple pericarp of papaya is formed.

## 2. Results

### 2.1. Sequencing Data Analysis of 12 RNA Libraries from Different Colored Papaya Pericarp

**Figure 1:**
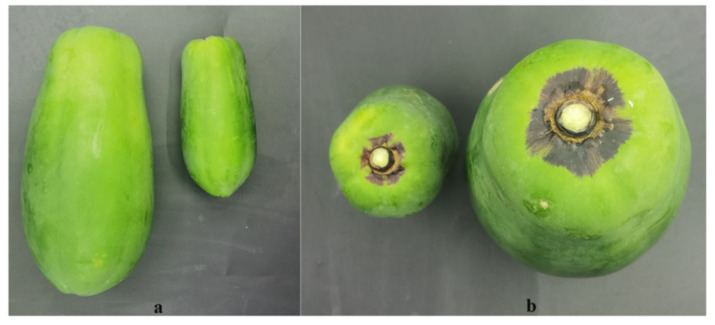
Phenotype of ‘Zihui’ papaya fruit in two developmental stages, 60 and 120 days after anthesis (60DAA, 120DAA). The fruit pericarp of green and purple parts for 60DAA and 120DAA were named YG, YP, MG, and MP, respectively. (**a**) Indicates fruit length, (**b**) reflects the fruit longitudinal diameter of the fruit.

RNA sequencing of the YG, YP, MG, and MP generated approximately 71.74 GB of nucleotide data (Figure 1). We obtained 38,351,276–43,072,194 reads for the samples, and 85.83 to 88.60% were mapped to the papaya reference genome. Scores greater than Q30 were found in over 93.73% of the clean data, and GC content ranged from 44.28 to 45.23%. The detailed information about RNA sequencing and mapping is summarized in Table 1.

**Figure 2:**
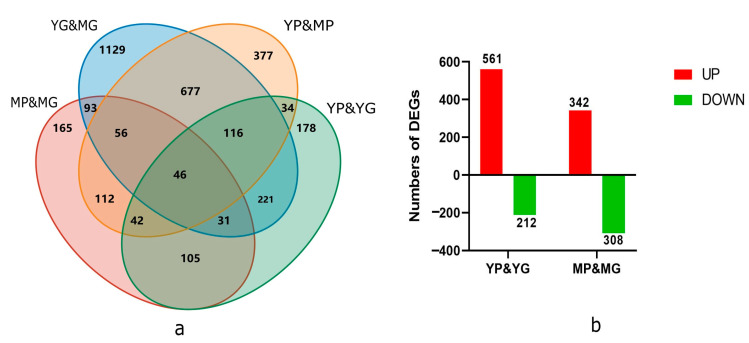
Number of DEGS in the transcriptomic profile of ‘Zhihui’ papaya pericarp samples. (**a**) Venn diagrams showing DEGs between YP&YG, YP&MP, YG&MG, and MP&MG. (**b**) Column charts of up-regulated and down-regulated DEGs from YP&YG and MP&MG.

Between the three biological replications, a high correlation was observed, indicating a steady expression pattern in the replicated samples (Figure S1). In total, 3382 DEGs were discovered in the comparison of YP&YG, YP&MP, YG&MG, and MP&MG. These genes included 773 (561 up-regulated and 212 down-regulated) and 650 DEGs (342 up-regulated and 308 down-regulated) between the YP&YG and MP&MG, respectively (Figure 2).

**Figure 3:**
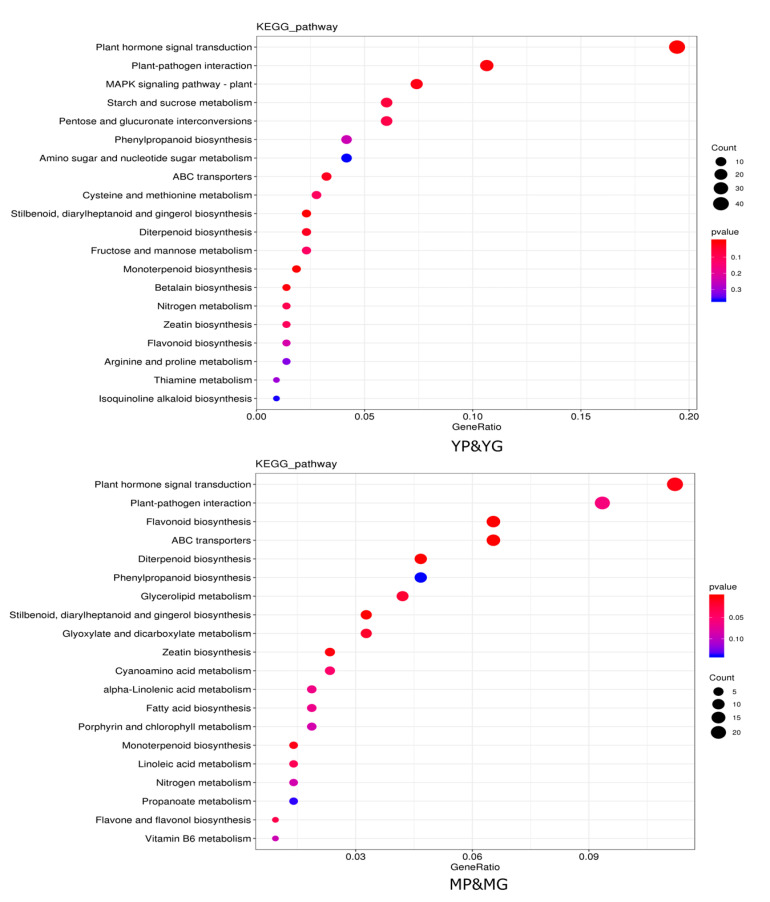
Enriched KEGG pathways of target genes for DEGS between YP&YG and MP&MG. Hotter colors correspond to lower *p*-values. The size of the circle indicates the number of DEGs.

The DEGs were performed with KEGG enrichment to better understand their biological functions; DEGs between YP&YG were mainly located in plant hormone signal transduction, plant–pathogen interaction, MAPK signaling pathway—plant, starch and sucrose metabolism, pentose and glucuronate interconversions pathways, etc. DEGs between MP&MG were mainly classified into plant hormone signal transduction, plant–pathogen interaction, flavonoid biosynthesis, ABC transporters, diterpenoid biosynthesis pathways, etc. Among them, DEGs between YP&YG and MP&MG were commonly enriched in plant hormone signal transduction, plant–pathogen interaction, ABC transporters, and flavonoid biosynthesis pathways, which indicated that those pathways may contribute to the formation of purple ring spots on the pericarp of papaya fruit tips (Figure 3).

### 2.2. Different Concentrations of Plant Hormones between Purple and Green Papaya Pericarp

To determine which plant hormones are responsible for the formation of purple ring spots on the pericarp of papaya fruit tips, a UHPLC-MRM-MS method was used to detect 24 target metabolites of plant hormones between MP and MG. Among the 24 target plant hormones, 11 were detected. Only four plant hormones, abscisic acid, 1-Aminocyclopropanecarboxylic acid, N-((-)-jasmonoyl)-S-isoleucine, and trans-Zeatin, were differentially expressed between the MP and MG (Table 2). 1-Aminocyclopropanecarboxylic acid and N-((-)-jasmonoyl)-S-isoleucine were up-regulated, while abscisic acid and trans-Zeatin were down-regulated in the MG pericarp. With RNA sequencing, there were 24 DEGs involved in signal transduction in plant hormones according to KEGG enrichment analysis. Among them, 15 DEGs were up-regulated, while nine DEGs were down-regulated in the MP group. The expression profiles of these GEGs are shown in Figure S2.

### 2.3. Metabolome Profiling of the Papaya Pericarp Related to Anthocyanin Metabolism

**Figure 4:**
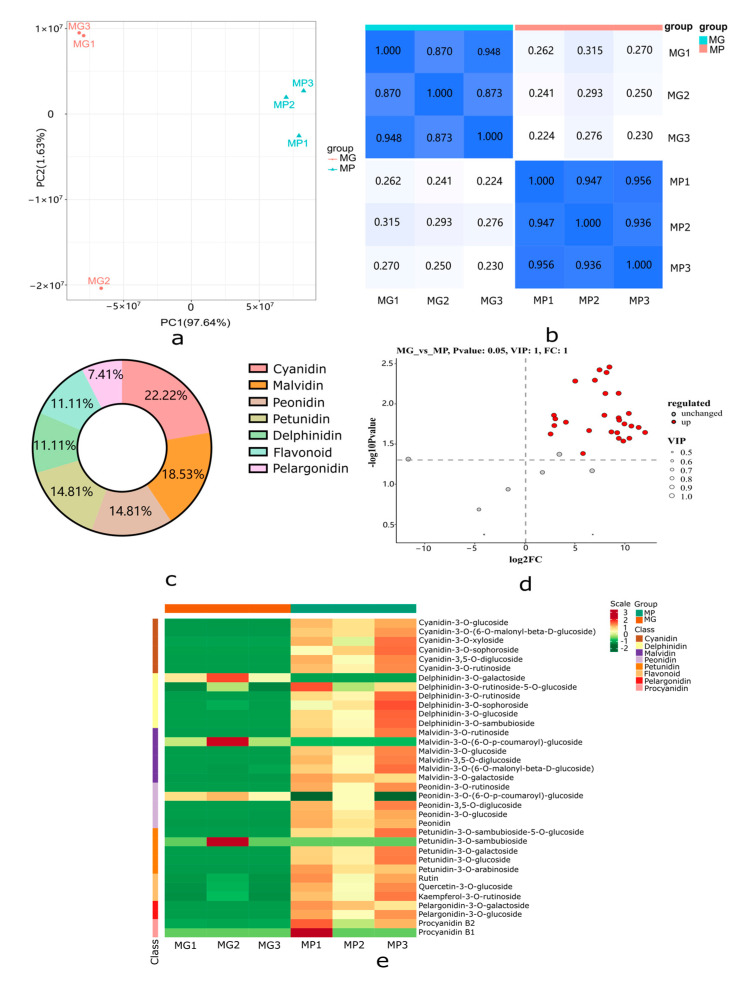
Metabolome profiling of MP and MG papaya pericarp related to anthocyanin metabolism. (**a**) Principal component analysis (PCA) score plots for MP and MG groups. (**b**) Spearman rank correlation for the metabolite determination results among different samples; the darker the color, the higher the correlation coefficient, while the lighter color indicate the lower correlation coefficient. (**c**) Primary classification of the DAMs identified between MP and MG groups. (**d**) Up-regulated and unchanged metabolite numbers between MP and MG groups; red dots indicate up-regulated metabolites and gray dots indicate unchanged metabolites. (**e**) Cluster heat map of the 35 anthocyanins in the MP and MG groups. Intensity values were adjusted by log transformation and then normalized.

To study the relationship between anthocyanin metabolism and the formation of purple ring spots on papaya pericarp, UPLC-MS-MS was used to detect 108 anthocyanins based on the public metabolic database, and 35 anthocyanins were detected between the MP and MG papaya pericarp. Anthocyanin derivative accumulations at the metabolomic level were responsive to color differences in papaya pericarp based on the PCA analysis (97.64%) (Figure 4a). Biological repeat correlation was evaluated using the Spearman rank correlation coefficient r. The closer r^2^ is to 1, the stronger the correlation between two duplicate samples. In this study, the intra-group correlation coefficients of the two samples were greater than 0.85, indicating the differential accumulation metabolites (DAMs) obtained were reliable (Figure 4b).

A heatmap cluster analysis of the 35 anthocyanin metabolites is shown in Figure 4e. Metabolites with |log_2_(fold change) ≥ 1|, *p*-value < 0.05, and VIP ≥ 1 were considered DAMs; 27 metabolites were significantly different in the MP and MG papaya pericarp (Figure 4d). All of the DAMs were up-regulated and grouped into seven categories in the MP group, including six metabolites in the largest category cyanidin, followed by malvidin (*n* = 5), peonidin (*n* = 4), petunidin (*n* = 4), delphinidin (*n* = 3), flavonoid (*n* = 3), and pelargonidin (*n* = 2) (Figure 4c; Table S2).

### 2.4. Integrated Analysis of DAMs and DEGs in Response to Different Colors of Papaya Pericarp

**Figure 5:**
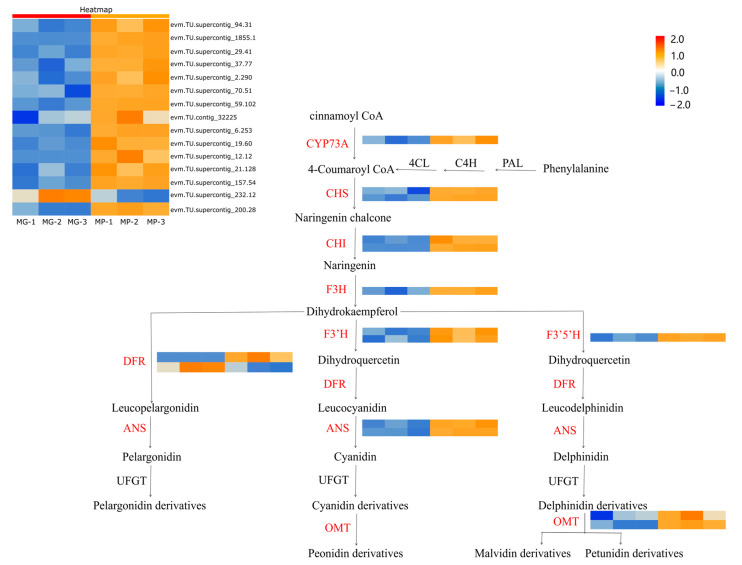
Analysis of DEGs related to the putative anthocyanidin biosynthesis pathway in *Carica papaya* L. Heatmap analysis of all DEGs was according to the FPKM value standardized by the Z-score of each gene. PAL (phenylalanine lyase); C4H (cinnamate 4-hydroxylase); CHS (chalcone synthase); 4CL (4-coumadin CoA ligase); CHI (chalcone isomerase); F3H (flavanone 3-hydroxylase); F3′H (flavanone 3′-hydroxylase); DFR (dihydroflavonol 4-reductase); ANS (anthocyanin synthase); UFGT (UDP-glucose flavonoid 3-glucosyltransferase); OMT (O-methyltransferase).

Analysis of combined transcriptomic/metabolomic regulatory networks was conducted to explore the differences in anthocyanin biosynthesis between MP and MG papaya pericarp. Flavonoid biosynthesis appeared to be the DEGs’ active biological pathway based on KEGG enrichment analysis. Fifteen DEGs were found on the flavonoid pathway, with two CHS (evm.TU.supercontig_70.51, evm.TU.supercontig_59.102), two CHI (evm.TU.supercontig_19.60, evm.TU.supercontig_1855.1), one F3H (evm.TU.supercontig_37.77), one F3′5′H (evm.TU.supercontig_157.54), one F3′H (evm.TU.supercontig_94.31), two DFR (evm.TU.supercontig_12.12, evm.TU.supercontig_232.12), two ANS (evm.TU.supercontig_6.253, evm.TU.supercontig_29.41), two OMT (evm.TU.supercontig_200.28, evm.TU.contig_32225), and one CYP73A (evm.TU.supercontig_2.290). From the heatmap (Figure 5), the CHS, CHI, F3H, F3′5′H, F3′H, ANS, OMT, and CYP73A were up-regulated in the MP group, and only one DFR was down-regulated. The high expression of these genes suggested that there was a greater amount of precursor compounds present in the MP group for anthocyanin synthesis. The metabolic profiling results showed significant differences in the intermediate products of anthocyanin biosynthesis corroborating this.

### 2.5. Correlation Analysis between Transcripts and Anthocyanidins

**Figure 6:**
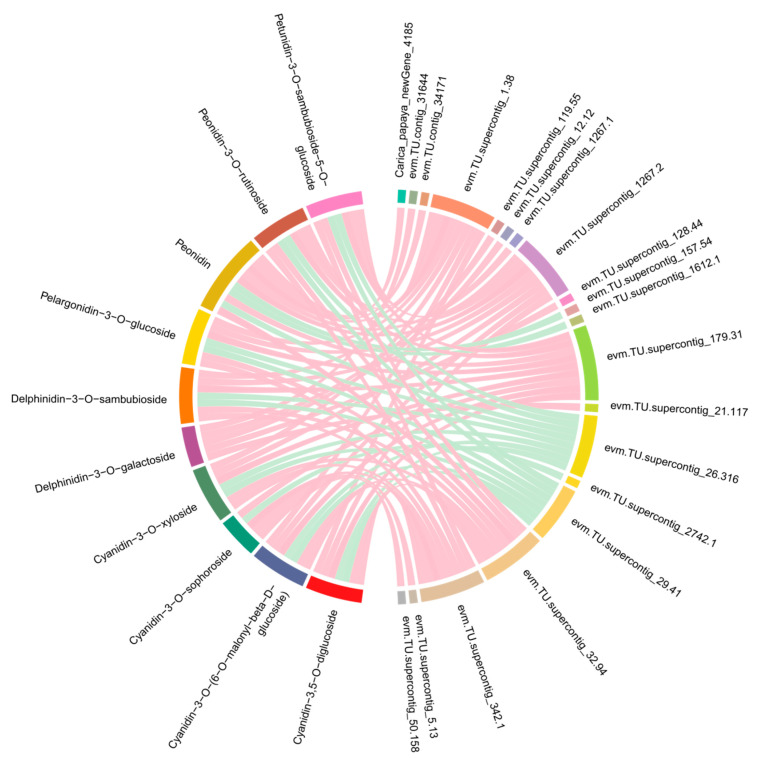
Correlation networks of the DEGs and DAMs involved in the anthocyanin biosynthesis pathway. The DEGs and DAMs are on the right and left of the figure, respectively. As shown by the pink line, the correlation was highly positive, while the cyan line represented a highly negative correlation.

**Figure 7:**
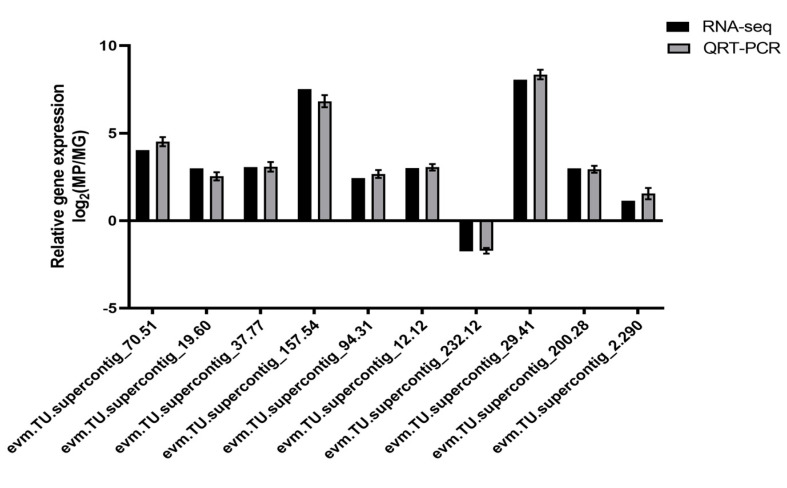
qRT-PCR verification of DEG relative expression levels. Standard deviations of three biological replicates are shown in the error bars. An evaluation of 10 DEGs using qRT-PCR and RNA-seq is shown in the gray and black bars, respectively.

It was still not clarified how those genes control the synthesis of anthocyanins in *C. papaya*. A co-expression network analysis of DEGs and DAMs was conducted to investigate the regulatory network of anthocyanidin biosynthesis in the different colored papaya pericarps. Twenty genes that had a high correlation (r > 0.9) with 10 anthocyanins, including nine structural genes, were identified: three OMT, three cytochrome P450 genes (CYP), one ANS, one DFR, one F3′5′H, four transcription factors (three MYBs and one SRF), and one WAT-related protein, one glutathione S-transferase F12 (GSTF12), one alpha carbonic anhydrase 1 (ACA1), one expansion-like B1 (EXLB1), one ribulose bisphosphate carboxylase small chain (RBCS), and two unknown proteins (Figure 6 and Table S3). MYB transcription factors play a crucial role in the synthesis of anthocyanins, and previous studies found that glutathione S-transferase was involved in flavonoid transport [29]. In our study, two MYB transcription factors, evm.TU.supercontig_119.55 (MYB5) and evm.TU.supercontig_32.94 (MYB-CC) had a highly positive relationship with delphinidin-3-O-galactoside and six anthocyanidin derivatives (cyanidin-3,5-O-diglucoside, peonidin, cyanidin-3-O-xyloside, peonidin-3-O-rutinoside, cyanidin-3-O-[6-O-malonyl-beta-D-glucoside], petunidin-3-O-sambubioside-5-O-glucoside), respectively. Evm.TU.supercontig_2742.1 (MYB113) was highly negatively related to cyanidin-3-O-sophoroside. The WAT-related protein, GSTF12, ACA1, EXLB1, and RBCS in this study were reported to be involved in anthocyanin biosynthesis in *C. papaya* for the first time. In summary, the above genes may play an important role in anthocyanin biosynthesis. To validate the transcriptome sequencing results, 10 genes were selected for qRT-PCR validation. As expected, these results coincided with transcriptome sequencing results (Figure 7).

## 3. Discussion

Papaya is a commercially popular tropical fruit tree with fruit rich in various vitamins and minerals considered beneficial for human health. Under normal conditions, the papaya fruit pericarp is green before ripening and orange-yellow when matured. In our study, the cultivar ‘Zhihui’ is special, with a circle of purple ring spots on the fruit tip that seriously affects its appearance. To date, there are few research reports on the color of the papaya fruit pericarp. One reason for this is the lack of diversity in research materials. This special material as the object of study can provide new insight into how the circle of purple ring spots on the fruit tip pericarp is formed [30].

Anthocyanins are part of flavonoids, which are water-soluble pigments that endow flowers, fruits, and seeds among several plant species with different colors [31]. The components of anthocyanins in various purple plant tissues have been identified in recent years. Compared to the yellow pericarp of passion fruits, the purple pericarp passion fruits accumulate more anthocyanins such as cyanidin 3-O-galactoside, peonidin 3-O-glucoside, and kuromanin [32]. Cyanidin-3-glucoside and peonidin-3-O-glucoside were considered the dominant anthocyanins in the purple rice grain [33]. Anthocyanins enriched in purple maize grain include peonidin-3-O-glucoside, cyanidin-3-O-glucoside, and pelargonidin-3-O-glucoside [34]. It seems that cyanidin-3-O-glucoside and delphinidin-3-O-glucoside are the main anthocyanins in purple barley grains [35]. Through the previous studies, although the components of anthocyanins were different in purple tissue among different species, a majority of purple plant tissues accumulated cyanidin-3-O-glucosides and peonidin-3-O-glucosides. In our study, 35 anthocyanins were detected between MP and MG papaya pericarp, and 27 anthocyanins were higher in the MP papaya pericarp. Among them, malvidin-3-O-rutinoside, delphinidin-3-O-glucoside, cyanidin-3-O-glucoside, peonidin-3-O-glucoside, malvidin-3-O-glucoside, petunidin-3-O-galactoside, peonidin-3-O-glucoside, and petunidin-3-O-glucoside were significantly accumulated in the MP papaya pericarp. The significant accumulation of cyanidin-3-O-glucoside and peonidin-3-O-glucoside was consistent with the above results [32,33,34,35]. Until now, there have been few reports on the color of the papaya pericarp, and this result can provide a reference for the formation of the purple color in papaya.

Plant hormones are key intrinsic environmental factors that play a prominent role in the regulation of plant anthocyanin signaling pathways [36]. Previous studies showed that low auxin levels can promote the expression of anthocyanin-related genes, thus promoting the accumulation of anthocyanins, while a high concentration of auxin inhibits it [37,38]. On the effect of ethylene treatment on photoinduced anthocyanin synthesis in sorghum, it was found that ethylene could promote anthocyanin accumulation in the early lagging stage but inhibit it in the late lagging stage [39]. Those results indicated that both auxin and ethylene have positive and negative regulation roles in the anthocyanin signal pathway; auxin is presented as an enhancement at low concentrations but is inhibitory at high concentrations. Anthocyanin accumulation has been demonstrated in rice, ornamental cabbage, and wheat after ABA treatment [40,41,42]. Cytokinin (CTK) can enhance the induction effect of light and can promote anthocyanin accumulation in the aleurone layer. This was also confirmed in rice and *Arabidopsis thaliana* [43,44]. It was found that salicylic acid (SA) could induce the expression of the ubiquitin-conjugating enzyme (UBC) OgUBC1 gene, and over-expression of OgUBC1 in *A. thaliana* increased anthocyanin accumulation and resistance to UV radiation and pathogens [45]. Through biological or abiotic stress stimulation, the level of endogenous jasmonic acid (JA) in plants was increased, and the genes related to JA and the anthocyanin synthesis signal were significantly up-regulated, resulting in anthocyanin accumulation [46]. The addition of exogenous gibberellin (GA) could significantly inhibit the accumulation of anthocyanins, while inhibition of gibberellin synthesis can increase the anthocyanin content in mature embryos [47]. In conclusion, plant hormones play different roles in regulating the anthocyanin signaling pathway in plants. Auxin and ethylene have both positive and negative regulatory effects; ABA, CTK, SA, and JA have positive regulatory effects, and GA has negative regulatory effects.

In our study, 11 plant hormones were detected by UHPLC-MRM-MS. Only four plant hormones, abscisic acid, 1-Aminocyclopropanecarboxylic acid, N-((-)-jasmonoyl)-S-isoleucine, and trans-Zeatin were differentially expressed between the MP and MG. Abscisic acid and trans-Zeatin were up-regulated in the MP pericarp, consistent with the result that ABA and CTK could promote the accumulation of anthocyanins. 1-Aminocyclopropanecarboxylic acid and N-((-)-jasmonoyl)-S-isoleucine were down-regulated in the MP pericarp, differently from the others, indicating that the pathways of plant hormones regulating anthocyanin synthesis vary among different plants. The analysis of its regulatory pathway and regulatory network requires further study.

In previous studies, two types of genes were reportedly associated with anthocyanidin biosynthesis: one type encodes enzymes (structural genes) that catalyze the synthesis of different anthocyanin glycosides in the flavonoid synthesis pathway, and the other type gene is regulatory and mainly includes transcription factors [48]. By combining transcriptome and metabolome profiling analyses, 15 structural DEGs related to the pathway of anthocyanin synthesis were identified, including two CHS, two CHI, one F3H, one F3′5′H, one F3′H, and two DFR, two ANS, two OMT, and one CYP73A. A positive correlation was found between anthocyanin accumulation and structural gene expression in fruit trees. It was determined that the structural genes *SmCHS*, *SmCHI*, *SmF3H*, *SmF3′5′H*, *SmDFR*, and *SmANS* displayed a higher abundance in purple eggplant pericarp compared with white eggplant pericarp [49]. A similar conclusion was also obtained from wild-type purple eggplants and mutants with other colors treated by EMS. This indicated that structural genes play a key role in the biosynthesis of anthocyanin in purple eggplant pericarp [50]. The genes responsible for anthocyanin synthesis structure, including CHS, F3H, F3′H, DER, and ANS, consistently exhibit higher levels of expression in purple plant tissue, resulting in significantly higher anthocyanin content compared to green or white tissue [51,52,53,54]. In our study, 14 anthocyanin synthesis structural genes were up-regulated in the MP group, and only one DFR was down-regulated. This result was consistent with previous studies [49,50,51,52,53,54], indicating that those genes were closely related to the formation of purple ring spots.

Anthocyanin synthesis structural genes directly participate in anthocyanin synthesis. In contrast, anthocyanin biosynthesis genes were found to be regulated by transcription factors MYBs. Three MYBs played a significant role in the anthocyanin synthesis pathway through a co-expression network analysis of DEGs and DAMs. The majority of MYBs could promote the biosynthesis of anthocyanins, and overexpression of AN1 and MYBA led to the up-regulation of CHS, F3′H, and DFR. This resulted in the accumulation of anthocyanins as reported, respectively, in purple potato and purple pepper [55,56]. MYB2 and Pr (MYB transcription factors) up-regulate the expression of downstream structural genes DFR, ANS, and UFGT in purple cabbage and purple cauliflower, resulting in purple leaves [57,58]. In addition, the transcription factor MYB plays a tissue-specific role in anthocyanin synthesis regulation, and different organs may be regulated by different MYBs. MdMYB1, MdMYBA, MdMYB10, and MYB110a were the key positive regulatory factors regulating anthocyanin synthesis in various tissues [59].

To maintain the metabolic equilibrium of anthocyanins in plants, certain MYBs act as negative regulators in anthocyanin biosynthesis. These MYBs are characterized by the presence of a repressor domain at the carboxyl end of the protein that hinders the transcription of genes associated with anthocyanin synthesis and negatively affects the production of anthocyanins [60,61,62]. In our study, MYB5 and MYB113 were up-regulated in the purple pericarp, while MYB-CC was down-regulated. MYB113 has been reported to have a positive correlation with anthocyanin accumulation in other plants [63,64,65]. The highly expressed BjMYB113 regulated purple leaves by enhancing the expression of anthocyanin biosynthesis genes [66]. To date, there have been no reports of MYB regulating anthocyanin synthesis in papaya. We hypothesize that MYB5 and MYB113 serve as key positive regulatory factors that enhance anthocyanin synthesis gene expression, while MYB-CC acts as a negative regulatory factor inhibiting the expression of anthocyanin biosynthesis genes. As a result of these findings, we speculate that purple ring spots on papaya fruit tips are formed when anthocyanins accumulate in the purple pericarp.

## 4. Materials and Methods

### 4.1. Plant Materials

The papaya variety ‘Zihui’ was used as the research material. Papaya fruits were sampled in two developmental stages, 60 and 120 days after anthesis (60DAA, 120DAA). The pericarps from the fruits of six individual plants were collected, pooled, and then immediately frozen and stored in liquid nitrogen for transcriptome and metabolome analysis. The biological replicates were conducted three times for each stage. The fruit pericarp of the green and purple parts for 60DAA and 120DAA were named YG, YP, MG, and MP, respectively. The fruits of ‘Zihui’ were planted in the Baiyun district of Guangzhou, Guangdong, China. Field management was the same as for the local papaya materials.

### 4.2. RNA Sequencing and Data Analyses

The total RNA of pericarp samples was extracted with three biological replicates using a FastQuant RT Kit (Takara, Dalian, China) following the manufacturer’s protocol. NanoDrop 2000, Qubit 2.0, Agilent 2100, etc., were used to assess RNA sample purity, concentration, and integrity. The RNA samples that met the requirements (concentration greater than 100 ng·uL^−1^) were used to construct an RNA library, as previously described [67], and then subjected to an Illumina sequencing platform (Illumina HiSeq X-ten, Illumina, Foster, CA, USA) to generate 150 bp paired-end reads. Fastq format was used to store the raw data. To obtain clean reads for further analysis, reads with poly-N, low quality, and less than 150 bp in length were filtered. HISAT2 version 2.0.5 was then used to align the clean reads to the papaya reference genome (http://www.ncbi.nlm.nih.gov/gene/?term=papaya, accessed on 12 August 2022).

### 4.3. Differentially Expressed Gene Identification

We calculated gene expression abundance based on fragments per kilobase of transcript per million fragments (FPKM), which were mapped according to gene length. Genes that were differentially expressed (DEGs) were identified by the DESeq R package, with a standard of |log_2_(foldchange)| ≥ 1 as well as a false discovery rate (FDR) < 0.05. The enrichment analysis (*p*-value ≤ 0.05) was performed based on the Gene Ontology (GO) terms and the Kyoto Encyclopedia of Genes and Genomes (KEGG) pathway.

### 4.4. Metabolites Extraction

The samples were freeze-dried by Scientz-100F and ground into powder (30 Hz, 1.5 min). A total of 50mg of each individual sample was accurately weighed and transferred to an Eppendorf tube. Then, 500 μL of extract solution (methanol/water/hydrochloric acid (500:500:1, V/V/V) was added, and after the sample was dissolved, the mixture was vortex oscillated once every 30 min for a total of 5 times to improve the extraction rate and refrigerated overnight at 4 °C. The extraction was then performed, as previously described [68].

### 4.5. UPLC-MS-MS Analysis

A UPLC-ESI-MS/MS system was used to analyze the sample extracts described previously [69]. Anthocyanins were analyzed using scheduled multiple reaction monitoring (MRM). Those metabolites with multiple |log_2_(fold change)| greater than 1, a *p*-value less than 0.05, and variable importance in the project (VIP) greater than 1 were considered differentially accumulated metabolites (DAMs).

### 4.6. Quantitative Reverse-Transcriptase PCR Validation

Total RNA of the pericarp samples was extracted using a FastQuant RT Kit (Takara) following the manufacturer’s instructions. Ten DEGs were selected for quantitative reverse-transcriptase PCR (qRT-PCR) validation. Primers were designed using NCBI primer BLAST (http://www.ncbi.nlm.nih.gov/tools/primer-blast/, accessed on 7 January 2023) and acted as the internal reference control. The qRT-PCR was performed using Bio-Rad’s CFX96 Real-Time System and the reactions program, following the method in the previous study [70]. The relative transcriptional levels were calculated using the 2^−ΔΔCt^ method. The primers are listed in Table S1.

## 5. Conclusions

In summary, we conducted transcriptome, plant hormone, and metabolome profiling analyses of green and purple pericarp, aiming to identify the potential mechanism of purple pericarp formation in papaya. In total, 650 DEGs were identified in the transcriptome sequencing between MG and MP, including 15 anthocyanin synthesis structure genes. Among these, 14 DEGs were up-regulated, except for a DFR in the purple pericarp that led to the accumulation of anthocyanins. Metabolome profiling found four plant hormones (especially abscisic acid and 1-Aminocyclopropanecarboxylic acid), and 27 anthocyanins showed significant differences in the MP and MG papaya pericarp. This result was consistent with the transcriptome analysis, which found all anthocyanins to be up-regulated. Moreover, three MYBs may regulate anthocyanin biosynthesis structure genes, crucial for anthocyanin synthesis. In addition to identifying numerous genes involved in the anthocyanin synthesis pathway of ‘Zihui’ papaya, this study offered new insights into the mechanism for purple pericarp formation.

## Figures and Tables

**Table 1:** Summary of RNA sequencing data.

Sample	Total Reads	Mapped Reads	Mapping Rate%	Unique Mapping Rate%	Multiple Mapping Reads%	GC%	Q30/%
MG-1	38,351,726	33,980,621	88.60	86.43	2.17	44.86	95.79
MG-2	42,073,822	36,849,432	87.58	85.40	2.18	44.68	95.07
MG-3	41,554,166	36,600,415	88.08	85.89	2.19	44.88	94.10
MP-1	41,276,198	36,194,284	87.69	85.83	1.86	44.81	93.73
MP-2	41,227,370	35,387,347	85.83	83.89	1.95	44.92	95.10
MP-3	41,212,436	36,040,076	87.45	85.32	2.13	44.43	95.01
YG-1	41,863,196	36,964,877	88.30	86.24	2.06	45.01	93.90
YG-2	41,476,052	36,522,639	88.06	86.19	1.86	44.29	95.10
YG-3	43,072,194	37,717,196	87.57	85.77	1.80	44.28	94.12
YP-1	41,486,398	36,277,749	87.44	85.25	2.20	45.23	93.97
YP-2	41,419,874	36,460,280	88.03	85.70	2.32	44.52	95.14
YP-3	41,542,488	36,476,648	87.81	85.45	2.35	44.81	94.83

**Table 2:** The comparison of contents of different plant hormones between MG and MP pericarp.

Plant Hormone Types	Metabolite Concentration (nmol/kg)	
	MG	MP
(+)-Abscisic acid	216.56 ± 65.88	2633.46 ± 194.76 *
1-Aminocyclopropanecarboxylic acid	2598.37 ± 396.12 *	0.00 ± 0.00
Salicylic acid	135.98 ± 23.66	218.01 ± 109.51
Indole-3-carboxaldehyde	99.94 ± 25.51	97.22 ± 7.43
Indole-3-acetic acid	82.21 ± 11.92	94.96 ± 20.32
N6-isopentenyladenosine	23.09 ± 8.43	27.83 ± 7.92
Dihydrojasmonic Acid	18.03 ± 1.74	13.49 ± 6.93
N-((-)-jasmonoyl)-S-isoleucine	14.97 ± 2.49 *	0.00 ± 0.00
trans-Zeatin-riboside	10.65 ± 1.79	16.20 ± 1.75
trans-Zeatin	1.02 ± 0.17	7.66 ± 2.02 *
N6-(delta 2-Isopentenyl)-adenine	1.15 ± 0.31	0.92 ± 0.13

* indicates significance at *p* < 0.05.

## Data Availability

Data are contained within the article or Supplementary Material.

## Supplementary Materials

The following supporting information can be downloaded at: https://www.mdpi.com/article/10.3390/molecules29071485/s1, Figure S1: Heat map of gene expression correlation between twelve samples; Figure S2: Expression profiling of differently expressed genes involved in plant hormone signaling in the pericarp of MG and MP groups; Table S1: Primers for quantitative real-time RT-PCR (qRT-PCR) validation; Table S2: Gene annotation of those DEGs that showed a high correlation with 10 anthocyanins; Table S3: Gene annotation of those DEGs that showed a high correlation with 10 anthocyanins.
