# Supplementary figures and images for: Transcriptome, Plant Hormone, and Metabolome Analysis Reveals the Mechanism of Purple Pericarp Formation in ‘Zihui’ Papaya (Carica papaya L.)
Source: Molecules. 2024 Mar 27;29(7):1485. doi: 10.3390/molecules29071485 (PMC11013584; doi:10.3390/molecules29071485)

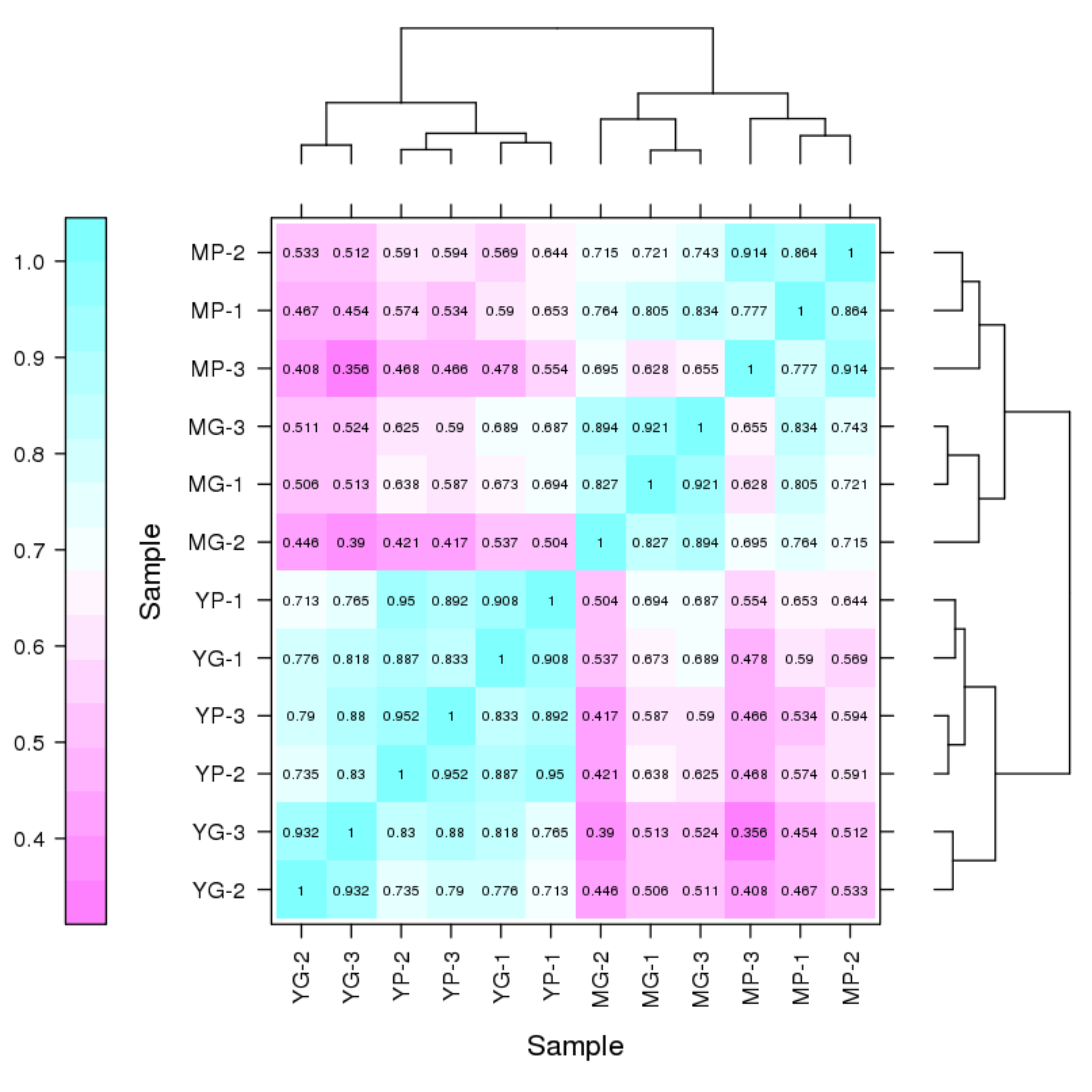

Supplement: Supplementary file 1 [file molecules-29-01485-s001.zip › Figure S1.tif]

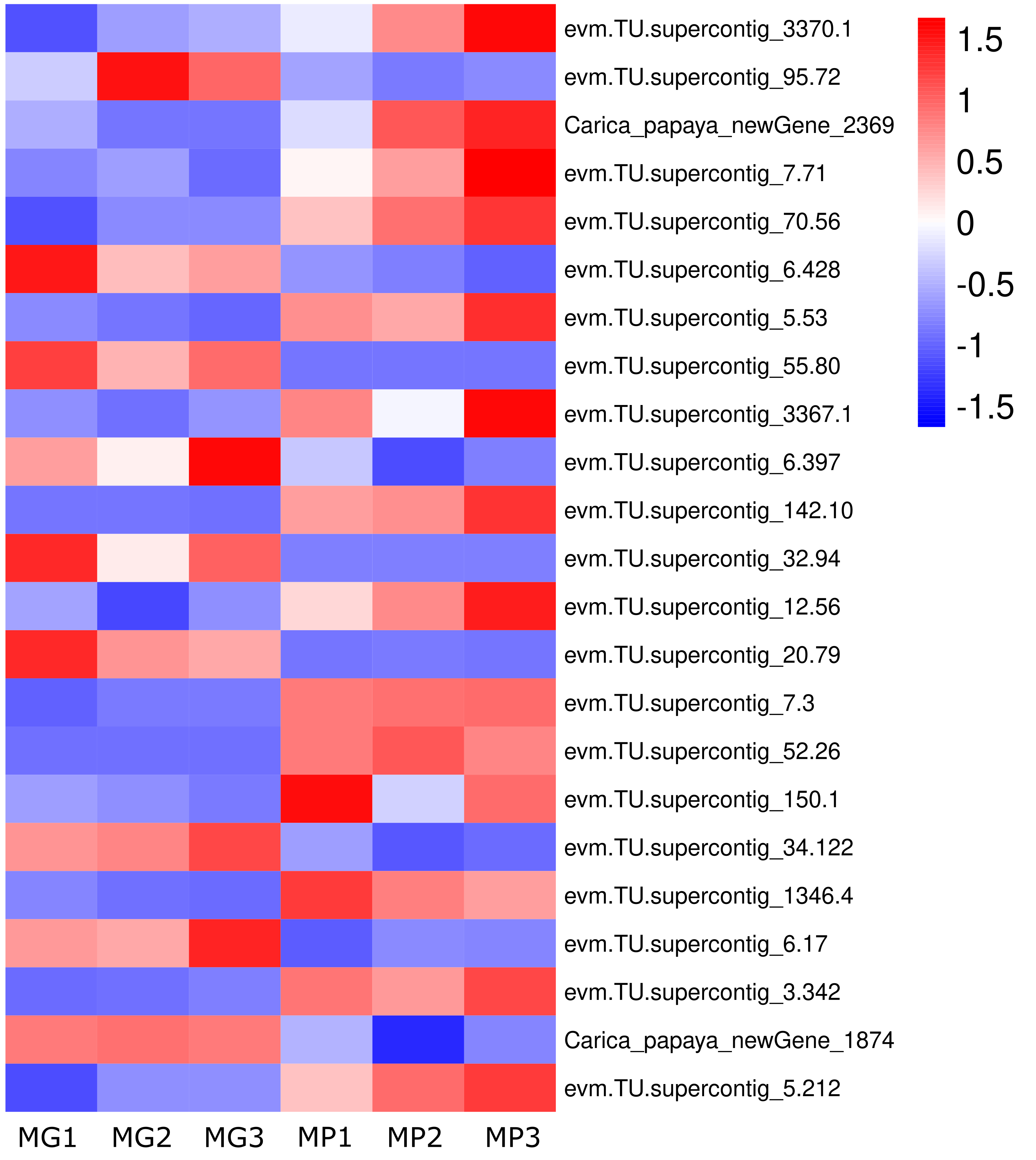

Supplement: Supplementary file 1 [file molecules-29-01485-s001.zip › Figure S2.tif]
